# Recent advances on 3D-printed PCL-based composite scaffolds for bone tissue engineering

**DOI:** 10.3389/fbioe.2023.1168504

**Published:** 2023-06-19

**Authors:** Maliheh Gharibshahian, Majid Salehi, Nima Beheshtizadeh, Mohammad Kamalabadi-Farahani, Amir Atashi, Mohammad-Sadegh Nourbakhsh, Morteza Alizadeh

**Affiliations:** ^1^ Student Research Committee, School of Medicine, Shahroud University of Medical Sciences, Shahroud, Iran; ^2^ Department of Tissue Engineering, School of Medicine, Shahroud University of Medical Sciences, Shahroud, Iran; ^3^ Tissue Engineering and Stem Cells Research Center, Shahroud University of Medical Sciences, Shahroud, Iran; ^4^ Regenerative Medicine Group (REMED), Universal Scientific Education and Research Network (USERN), Tehran, Iran; ^5^ Department of Tissue Engineering, School of Advanced Technologies in Medicine, Tehran University of Medical Sciences, Tehran, Iran; ^6^ Faculty of New Sciences and Technologies, Semnan University, Semnan, Iran

**Keywords:** bone tissue engineering, PCL composites, 3D printing, bone scaffolds, 3D printed PCL

## Abstract

Population ageing and various diseases have increased the demand for bone grafts in recent decades. Bone tissue engineering (BTE) using a three-dimensional (3D) scaffold helps to create a suitable microenvironment for cell proliferation and regeneration of damaged tissues or organs. The 3D printing technique is a beneficial tool in BTE scaffold fabrication with appropriate features such as spatial control of microarchitecture and scaffold composition, high efficiency, and high precision. Various biomaterials could be used in BTE applications. PCL, as a thermoplastic and linear aliphatic polyester, is one of the most widely used polymers in bone scaffold fabrication. High biocompatibility, low cost, easy processing, non-carcinogenicity, low immunogenicity, and a slow degradation rate make this semi-crystalline polymer suitable for use in load-bearing bones. Combining PCL with other biomaterials, drugs, growth factors, and cells has improved its properties and helped heal bone lesions. The integration of PCL composites with the new 3D printing method has made it a promising approach for the effective treatment of bone injuries. The purpose of this review is give a comprehensive overview of the role of printed PCL composite scaffolds in bone repair and the path ahead to enter the clinic. This study will investigate the types of 3D printing methods for making PCL composites and the optimal compounds for making PCL composites to accelerate bone healing.

## Graphical Abstract

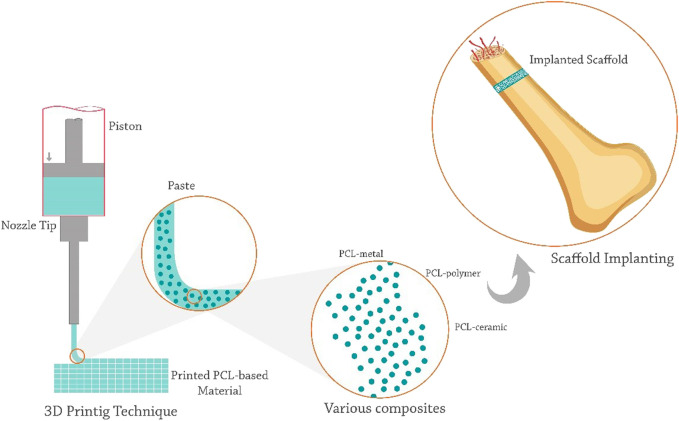

## 1 Introduction

Bone is a specialised connective tissue composed of a calcified extracellular matrix (ECM) and three main types of cells: osteocytes, osteoblasts, and osteoclasts (Rho et al., 1998). Bone tissue plays multiple roles in daily life, including storing and releasing minerals, supporting the body, enabling and facilitating movement, and protecting the body’s internal organs (L Mescher, 2018). Many people suffer from bone diseases caused by tumour resection, trauma, infection, cysts, congenital defects, and injuries caused by accidents (Li et al., 2020a; Biscaia et al., 2022a). Bones are dynamic and vascular organs that are regenerated and repaired throughout (Heo et al., 2017; Hassanajili et al., 2019a). Bone fracture repair occurs during the four phases of hematoma formation, soft callus formation, hard callus formation, and bone remodelling (Monfared et al., 2022).

Bone tissue has a strong potential to regenerate itself after injury; however, effective repair of large and critical bone defects still requires bone grafts (Carano and Filvaroff, 2003; Lanza et al., 2020). More than two million bone graft surgeries are performed each year, more than a quarter of which are performed in the United States (Campana et al., 2014; Buyuksungur et al., 2021a). Demand for bone grafts is expected to increase in the coming decades as the population ages (for example, in Germany, half of the population is over 45 and one-fifth is over 66) (Huber et al., 2022). Natural bone replacements have been widely used in clinical applications. Autograft is the most commonly used traditional option for patients with osteoporosis; however, complex issues such as donor site complications, infection risk, and a lack of bone grafts of appropriate size and shape have limited its use in orthopaedic applications (Keating et al., 2005; Rezania et al., 2022a). In addition, allograft will be a treatment option that uses a bone replacement from another person. Transmission of infection and disease, limited resources, insufficient integration with bone tissue, and the risk of immune rejection are also obstacles to the success of allografts (Fleming et al., 2000; Erbe et al., 2001).

Bone tissue engineering (BTE) is one of the best alternative methods to overcome these shortcomings, as it can be produced on a large scale without immune rejection (Hwang K.-S. et al., 2017). BTE focuses on key processes such as cell growth, customization of bone grafts, and minimization of the need for additional surgeries (Kim S. E. et al., 2016; Hwang K.-S. et al., 2017). It uses a three-dimensional (3D) scaffold to create the proper microenvironment for cell proliferation and regeneration of damaged tissues or organs (Biscaia et al., 2022a). In BTE, biomaterials are used alone or in combination with appropriate biological, chemical, and mineral agents to repair damaged bone tissue (Hernandez et al., 2017a).

An efficient 3D scaffold for BTE must possess unique features such as osteoinduction, osteoconduction, and osseointegration (Beheshtizadeh et al., 2021; Duan et al., 2021). Osteoconductivity of the scaffold helps eliminate the formation of fibrous capsules and creates a strong bond with the host bone (Wang C. et al., 2017; Zimmerling et al., 2021). These biomimetic scaffolds should possess biocompatibility and bioactivity to encourage appropriate cellular adhesion. Furthermore, these scaffolds should replicate bone structure, shape, and function (Park H. et al., 2018; Koons et al., 2020).

Scaffolds must also have optimal geometry and porosity, which are critical to preserving space for bone regeneration, supporting the periosteum, and filling the anatomical structure of bone defects (Ku et al., 2015; Giuliani et al., 2016; Koons et al., 2020). The interconnected shape of macro- and microporosity allows bone tissues and arteries to grow inside the scaffold and deliver nutrients and oxygen to the cells. Different pore sizes affect cell behavior, and pore sizes close to 300 μm are optimal for bone growth (Karageorgiou and Kaplan, 2005; Rezwan et al., 2006). Additionally, the scaffold needs to be mechanically strong enough to support the structural requirements of the tissue replacement, and its gradual degradation rate must be proportional with growth, cell proliferation, and new bone tissue formation. The scaffold must have an elastic modulus similar to human bone tissue (7 GPa–25 GPa) to prevent stress shielding (Pattanashetti et al., 2019; Fallah et al., 2022; Huber et al., 2022).

The used materials and the scaffold’s fabrication methods are key parameters in controlling its properties. Various traditional methods such as, solvent casting (Thadavirul et al., 2014), electrospinning (Metwally et al., 2020), and phase separation (Salerno and Domingo, 2015) have been used in the BTE approach. Since traditional techniques cannot precisely control the size, geometry, and interconnection of scaffold pores, in recent years, additive manufacturing methods [e.g., 3D printing (Liu Y. et al., 2020; Zimmerling et al., 2021)] have been considered as a promising approach to repairing critical bone defects in a clinical setting (Sachlos and Czernuszka, 2003; Wubneh et al., 2018).

A 3D printing technique possessing appropriate features, such as spatial control of micro-architectural and scaffold composition and high efficiency and accuracy, has become one of the top research topics in BTE (Galarraga et al., 2019; Wang et al., 2020; Wang F. et al., 2022; Wang P. et al., 2022). This technique has revolutionised scaffold fabrication and is generally regarded as the most symbolic tool of the third industrial revolution (Wibowo et al., 2020; Xu Z. et al., 2021).

Due to the diversity of the molecular weight, surface chemistry, and crystallinity of various biomaterials, the scaffold’s swelling, biocompatibility, degradability, and mechanical properties are varied (Burg et al., 2000; Rezwan et al., 2006). Therefore, choosing a suitable biomaterial is also quite essential. Various polymeric (Grémare et al., 2018), metal (Ahn et al., 2018), ceramic (Mondal et al., 2020), and composite (Farokhi et al., 2018) materials have been used to fabricate bone scaffolds. PCL is one of the most common materials in bone tissue regeneration (Wang C. et al., 2022). The slow degradation of PCL provides adequate time for bone regeneration and can also be manipulated to regulate the biodegradability rate of this polymer. Studies showed that PCL is completely degraded *in vivo* within 3–4 years after transplantation and has excellent bone graft stability and affinity (Xue et al., 2017; Gautam et al., 2021).

However, due to the hydrophobicity and lack of osteogenesis potential of PCL, researchers generally use PCL-based composite scaffolds [in combination with a variety of metal (Wang S. et al., 2022; Lei et al., 2022), polymer (Ke et al., 2022), and ceramic (Cao et al., 2022) materials] for tissue engineering applications to improve the mechanical and biological properties of the prepared constructs (Chen et al., 2004; Xiao et al., 2021).

This study highlights the opportunities and challenges of PCL-based 3D printed composite scaffolds for BTE and identifies the path ahead for them to enter the clinic. First, it introduces the types of 3D printing methods that have been used so far for PCL-based composite scaffolds for BTE and describes the parameters affecting the optimal design of the relevant inks. Then, it introduces the types of ceramic, polymer, and metal materials that have been used in combination with PCL and 3D printing for BTE. The later sections express the importance of the presence of drugs, growth factors, and cells in the above composites for bone repair. Finally, the clinical trials and the future path of these scaffolds to enter the clinic will be discuss.

## 2 Additive manufacturing techniques

The additive manufacturing (AM) technology mainly includes fused deposition modelling (FDM), binder jetting, directional energy deposition, material extrusion, selective laser sintering (SLS), material jetting, Sheet Lamination, Vat Photopolymerization, Powder Bed Fusion, and stereolithography (SLA) (Murphy and Atala, 2014; Turnbull et al., 2018; ASM International et al., 2020). AM produces objects through the successive layering of powders, liquid, or solid materials in accordance with a 3D design and the specified process parameters. The AM technology makes it possible to control the temporal and spatial distribution of inks (cells and biomaterials) and the spatial distance between them. Patient-specific computer-aided design (CAD) models can be created by converting computed tomography (CT) or magnetic resonance imaging (MRI) images of clinical defects to CAD models. Other facilities are then used to cut CAD models into G-code, which encrypts 3D CAD models into a format that can control the machine (Boschetto and Bottini, 2014; Mohamed et al., 2015; Zimmerling et al., 2021). Reproducibility, precise deposition, the creation of complex high-resolution 3D structures, cost-effectiveness, controlled morphology and size of pores, simplicity, and the ability to control cell distribution are just some of the advantages of AM methods (Haffner et al., 2018; El-Habashy et al., 2021).

The AM techniques allow the integration of vascular structures in tissue engineering constructs and thus overcome the challenges of nutrient transfer in scaffolds made with other methods (Hann et al., 2019). This approach seeks to develop innovative scaffolds that enhance the mechanical properties of BTE constructs in load-bearing applications (Farzadi et al., 2014; Wang L. et al., 2017). The AM increases the efficiency of tissue engineering scaffolds by using a wide range of materials and cells within the final structure. To achieve this, the biomaterials must possess a high rate of printability (Kyle et al., 2017; Zimmerling et al., 2021). The constructs obtained by AM facilities can be mixed with live cells before fabrication (in some techniques) or loaded with cells upon fabrication. A printed material may require post-processing procedures such as the removal of preservatives, surface modification, and sintering in order to mature and achieve the desired geometry and structure (Temple et al., 2014; Haffner et al., 2018).

The AM methods can adjust various properties of bone scaffolds, such as stiffness, the spatial distribution potential of biochemical factors, complex and irregular shapes, pore shapes and dimensions, and surface morphology (Cox et al., 2015; Wang et al., 2016a; Du et al., 2019). In addition, this technology allows for high cell density and *in vivo* interaction. Ink containing various cell types and ECMs can print large-scale damaged bone scaffolds through the high-density bioprinting techniques (Russmueller et al., 2015; Celikkin et al., 2022). The possibility of processing drugs and biomolecules using various materials, maintaining structure and shape, minimising material loss, improving mechanical properties, and increasing cell penetration and nutrient circulation have made the AM a desirable approach in BTE (Chen et al., 2020; Celikkin et al., 2022). A variety of materials could be used in AM strategies, including natural and synthetic materials. Meanwhile, PCL, a printable thermoplastic polymer (melting point 59°C–64 °C), is one of the first polymers used in this technique. For the first time in 2006, 3D-printed PCL scaffolds were able to receive FDA approval as a bone filler in skull and facial applications (FDA, 2006).

### 2.1 Various AM techniques used for PCL-based scaffold fabrication

**FIGURE 1:**
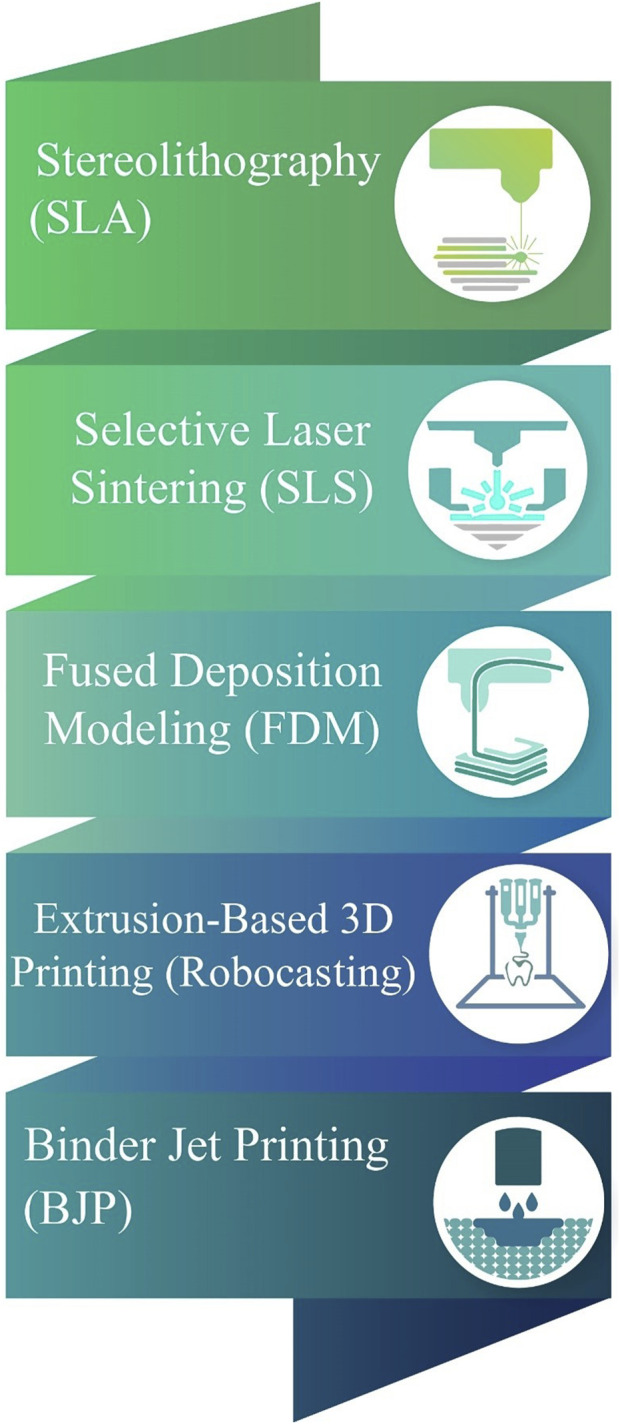
Various AM methods for fabricating PCL-based scaffolds.

The properties of the 3D printing scaffold are affected by the printing technology. AM techniques used in fabricating the PCL-based scaffolds include SLA (Elomaa et al., 2020), SLS (Eosoly et al., 2012), FDM (Bruyas et al., 2018a), and bioprinting (Koch et al., 2022) (Figure 1). These methods wrer summarized in Table 1.

**TABLE 1:** 3D printing methods for PCL-based scaffolds.

AM technique	Advantages	Disadvantages	Ref
FDM	Appropriate mechanical and biochemical properties for bone regeneration; available, user-friendly, reproducible	High temperature, limited resolution	Zhao et al. (2020a), Xu et al. (2021b)
SLS	Control pore size and porosity, non-load-bearing scaffolds	Expensive, high temperature, not able to create small pores	Partee et al. (2006), Liu et al. (2020b)
SLA	High accuracy, produce complex shapes, high resolution	Expensive, difficult to build micron-sized scaffolds, limited layer thickness, cytotoxicity caused by photoinitiator	Melchels et al. (2010), Elomaa et al. (2011a), Elomaa et al. (2020)
Binder-jet-based	Cheap, fast, cell survival rate of 85%, Compatible with various materials	Limited direction of injection, low resolution, low cell density	He, 2016; He et al. (2017)
Extrusion-based	Simple, inexpensive, cell survival rate of 40%–80%, tunable speed, high cell density	Slow, limited material	Pati et al. (2015a), Borkar et al. (2021)

FDM, or fused filament fabrication (FFF), is the preferred method for PCL deposition. PCL’s thermoplasticity has made it a desirable polymer for the available and uncomplicated FDM method (Arealis and Nikolaou, 2015; Xu X. et al., 2021). PCL filaments or small grains begin to melt by being placed in a temperature-controlled printing head, melt extrude of a small nozzle, and are layered on a platform to build a 3D structure (Xu et al., 2014; Arealis and Nikolaou, 2015). In the printing environment, the melt gradually cools and creats a scaffold with high reproducibility and controlled pores (Schantz et al., 2005). However, the high temperature of this method has limited the use of heat-sensitive biomolecules and polymers in combination with PCL.

In this method, the viscosity of the melted polymer restricts the resolution, shape, and even regularity of the scaffold (Zein et al., 2002; Zhao et al., 2020a). The PCL scaffolds obtained by this method have dimensions of hundreds microns (sometimes up to millimeters) (Xie et al., 2019). Zhao et al. (Zhao et al., 2020a) used the FDM method to print magnesium/PCL composite scaffolds. Their results showed that the resulting scaffold had suitable biocompatibility, mechanical properties, and osteogenesis. Using this method, they were able to evenly mix and print the optimal percentage of magnesium inside the scaffold. Rezania et al. (Rezania et al., 2022a) used this method to print hydroxyapatite/PCL scaffolds. Hydroxyapatite/PCL filaments showed that this method could be considered a commercially viable and suitable method for producing scaffolds with appropriate mechanical and biological properties for BTE applications.

SLS is a more expensive but more accurate method for fabricating PCL composite scaffolds. The SLS equipment has several parts, including a laser source, a powder bed, and a piston and roller to spread a new layer of powder (Williams et al., 2005; Partee et al., 2006). The laser beam (according to the pattern set by the computer) sinters certain parts of the powder bed and creates layer-by-layer PCL scaffolds.

SLS has been used to fabricate bioactive and composite PCL scaffolds with mechanical properties similar to trabecular bone. This method is suitable for making non-load-bearing PCL scaffolds (Williams et al., 2005; Doyle et al., 2015). However, due to the high temperature involved in the process, the inclusion of cells and biomaterials in SLS scaffolds is limited, and this method is only compatible with a limited number of materials (Liao et al., 2016; Liu et al., 2020b). Studies showed that SLS was not able to create a scaffold with small pores. Liu et al. (Liu et al., 2020b) fabricated PCL/hydroxyapatite scaffolds by SLS. The PCL/hydroxyapatite microspheres led to the construction of scaffolds with interconnected pores that supported cell proliferation and the penetration of blood vessels. In addition, The authors highlighted that the loading of VEGF onto the scaffolds enhanced angiogenesis and osteogenesis (Liu et al., 2020b).

SLA is one of the most expensive AM techniques, with high manufacturing accuracy and the ability to produce complex shapes with a relatively high resolution up to 1.2 μm, which uses light-activated biopolymers (Melchels et al., 2010; Ronca et al., 2021). SLA is one of the preferred printing methods, utilizing composites of photo-curable polymer (such as GelMA) in combination with PCL (Elomaa et al., 2020). The basis of SLA is light-sensitive ink photopolymerization, and the desired model is photocross-linked in each layer to form the desired 3D structure. However, due to the limited layer thickness, over-drying, and partial polymerization of the resin in the substrates, it is difficult to build micron-sized scaffolds by SLA. In addition, the presence of a photoinitiator and ultraviolet radiation sometimes causes cytotoxicity.

The cross-linking of patterns in this method does not apply shear stress to cells. The UV light source has high energy and speed for ink cross-linking, but due to its destructive effect on cell survival, the visible light source can also be used (Melchels et al., 2010; Elomaa et al., 2011a). Using solvent-free stereolithography, Elomaa et al. (Elomaa et al., 2011b) prepared a photo-curable PCL-based resin. The resulting scaffold had high accuracy, no shrinkage, and interconnected pores of suitable size and shape.

Due to the non-uniform distribution of cells on printed scaffolds, the bioprinting method allows the simultaneous deposition and uniform distribution of living cells, macromolecules, and other biomaterials within the structures (Cunniffe et al., 2017; Genova et al., 2020). It is preferred to use PCL in combination with hydrogels containing live cells for bioprinting PCL-based scaffolds. Bioprinters typically have several printing nozzles, one for printing polymers like PCL and another for printing cells and heat-sensitive materials. This method allows the integration of various cells [such as mesenchymal stem cells (MSCs) and human umbilical vein endothelial cells (HUVECs)] into scaffolds and induces vascular formation (Genova et al., 2020; Yang et al., 2021).

The binder-jet-based printing (He, 2016), and extrusion-based printing (Borkar et al., 2021) are also used in preparing the PCL composite scaffolds. It should be noted that the use of PCL alone is not recommended in bioprinting and encapsulation, as this polymer is a hydrophobic material with a destructive temperature and viscosity for cells and requires high pressure for printing. Hence, by using co-printing and its combination with suitable polymers, the desired properties of this polymer can also be used in bioprinting (Yu et al., 2014; Murphy et al., 2017a). Murphy et al. (Murphy et al., 2017a) fabricated a bioactive borate glass/PCL/matrigel composite containing human adipose stem cells using multi-nozzles bioprinting. Their results showed that the scaffolds had favorable bioactivity, cell survival, angiogenesis, and cellular interactions.

### 2.2 Optimizing 3D printing parameters

**FIGURE 2:**
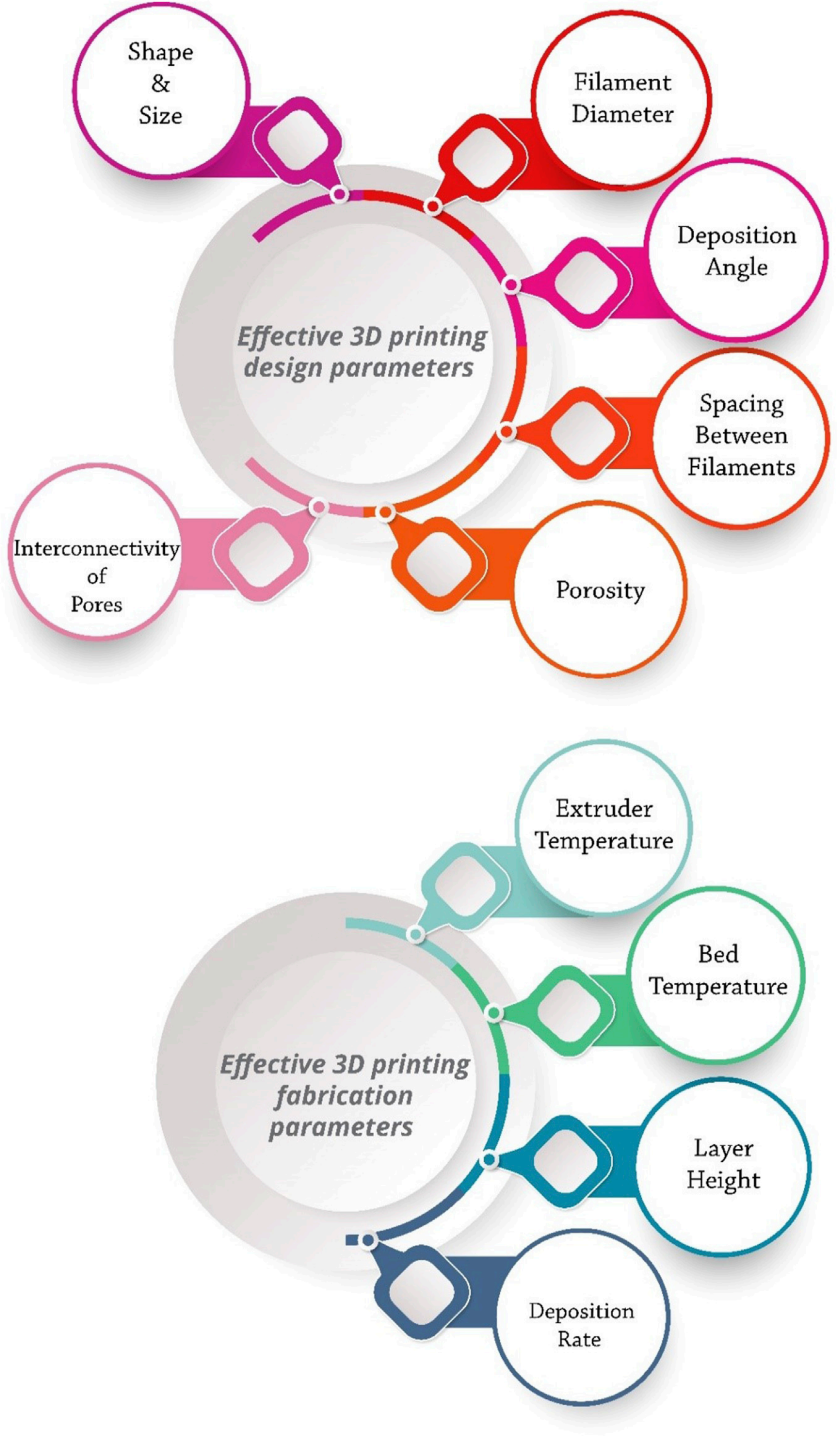
Effective 3D printing parameters for fabricating the PCL-based scaffolds.

Proper ink design, selection of suitable material for bone tissue engineering, dimensions of the final model, time required for modeling, and dimensional accuracy are the most significant challenges of bioprinting (Rabionet et al., 2018a; Midha et al., 2019). Ink should be selected in such a way that it simultaneously has the necessary mechanical and physiological properties for the printing process and the bone tissue (Liu et al., 2019). Developing specific and appropriate inks and optimizing printing parameters can significantly improve the treatment of bone defects and clinical outcomes (Figure 2).

Printing parameters include two categories: design and manufacturing. Design parameters include filament diameter, deposition angle, and spacing between filaments, which determine the overall scaffold architecture. Extruder temperature, bed temperature, layer height, and deposition rate also control the printing process as fabrication parameters (Rabionet et al., 2018a; Modi and Sahu, 2021). The shape, size, interconnectivity of pores, and porosity of the scaffold affect the osteogenesis and mechanical properties of the scaffold. By adjusting the parameters of the 3D printing method, various characteristics of the scaffold can be controlled and optimized, such as pore size and shape, porosity, mechanical properties, and cell behavior (Park S. A. et al., 2018). For example, circular pores have higher fatigue resistance than triangular pores (Gong et al., 2017). Five-angle scaffolds under compressive loading have less stiffness than three-angle scaffolds. Even the deposition angle of scaffold layers can affect cellular responses. For example, a study showed that 0°, 60°, and 120° stimulated cell proliferation better than 0°, 72°, 144°, 36°, and 108° in the first 2 weeks, but after three and 4 weeks, the results were quite the opposite (Rabionet et al., 2018b).

In BTE, there must be an optimal balance between pore size and the mechanical strength of the scaffold. The number and height of the printed layers are also quite influential. The layer’s height is inversely related to the printing speed and dimensional accuracy (Pazhouhnia et al., 2022). The thickness of each layer must be greater than the porogen particles to ensure proper bonding of the layers. On the other hand, excessive layer height leads to extensive z steps. The layer’s height is controlled by the nozzle’s diameter (Zhao D. et al., 2020; Modi and Sahu, 2021).

**FIGURE 3:**
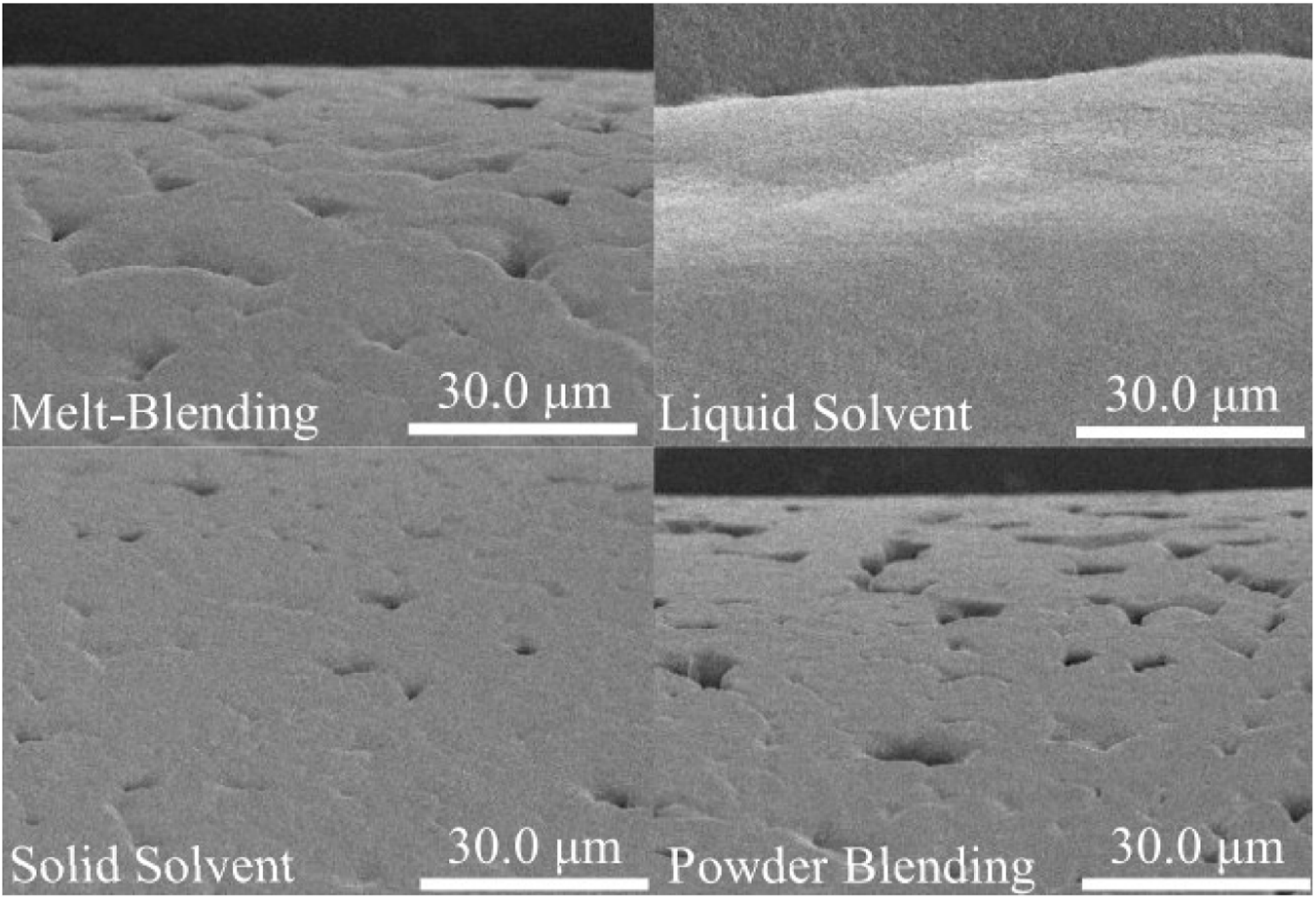
The effect of the preparation method on the SEM images of 3D-printed PCL-HAp filaments (1500X magnification), reprinted with permission from (Zimmerling et al., 2021). Scaffolds prepared with powder blending had the deepest and largest number of pores on the surface of the filaments, and scaffolds based on liquid solvent had a smooth surface due to the lower viscosity of the liquid and the filling of the pores by materials during printing.

In addition, various PCL-based ink preparation methods for printing also affect the printability, swelling/degradation, and mechanical properties of scaffolds. Melt-blending, powder blending, liquid solvent technique, and solid solvent technique are standard strategies for preparing PCL-based inks (Figure 3) (Zimmerling et al., 2021). The solvent or combination of solvents is a crucial factor in their scaffold properties. Various solvents have multiple interactions with PCL and have diverse volatility, protein conductivity, and dispersion of particles, resulting in individual uniformity and structural order.

## 3 PCL-based composites

PCL-based scaffolds were introduced more than decades ago for BTE (Beheshtizadeh et al., 2020). These scaffolds were implanted in more than 20,000 patients and helped repair bone defects (Teoh et al., 2019). As mentioned previously, PCL is a Food and Drug Administration (FDA)-approved linear thermoplastic and aliphatic polyester with high biocompatibility, low cost, easy processing, non-carcinogenicity, low immunogenicity, a slow degradation rate, and lower acid degradation products compared to other polyesters (Malikmammadov et al., 2018; Malikmammadov et al., 2019; Bikuna-Izagirre et al., 2022). This semi-crystalline polymer has sufficient potential for use in high-load-bearing bones (Lee et al., 2011).

Despite the beneficial properties mentioned for PCL, its hydrophobicity, low bioactivity, and slow degradation rate remain major challenges in biomedical applications. Mixing or copolymerizing PCL with other components leads to altered mechanical, surface, and physicochemical properties of the scaffold. In this regard, various research has used the combination of diverse metals, ceramics, and polymers with PCL to improve its features, which will be discussed in detail in this section (Table 2).

**TABLE 2:** Some recent studies on PCL-based composites in BTE applications.

	Material	Printing parameters	Animal model	Cell/biomolecule	Outcomes	Ref
PCL/ceramic composites	PCL	• Extrusion-based technique	Rat, tibial defect (8 mm)	Alendronate	• Sustained release of alendronate	Kim et al. (2016a)
		• Liquid solvent technique			• Improved ALP activity and calcium deposition • Increased new bone formation and mineralization	
		• Speed and air pressure: 5 mm s−1 and 80 kPa				
		• Nozzle size: 25-G				
	PCL/Strontium- and cobalt- doped bioactive glass	• 30% porosity	-		• PCL/Strontium- and cobalt- doped bioactive glass improve Young’s modulus, apatite-forming ability, and cytocompatibility	Fathi et al. (2020)
		• Nozzle size and temperature: 500 μm and 120 °C				
		• spacing between the filaments: 400 μm				
	PCL/nanoparticulate Willemite	• Liquid solvent technique	Rabbit, Femoral defect (Osteonecrosis model)		• PCL/npW improved cytocompatibility, osteogenic activity, and HA accumulation	Karimzadeh Bardeei et al. (2021)
		• Temperature 75°C, pressure 3.38 bar				
		• Speed 6 mm/min				
	PvCL/Bioactive borate glasses	• Extrusion-based Liquid solvent technique	-	Adipose stem cells	• Improve angiogenesis and bioactivity	Murphy et al. (2017a)
		• Scaffolds 10 × 10 × 1 mm^3^			• Sustained release of bioactive glass	
		• pore sizes 100—300 μm			• 60% cell viability	
		• pressure 10–50 psi, nozzle size 110–600μm				
	PCL/HA	• Extrusion-based technique	-	-	• The mechanical characteristics and crystallinity of the scaffold were improved by HA. • In comparison to solvent casting ink preparation, melt blending ink preparation has improved mechanical qualities, cytocompatibility, and osteogenic potential	Biscaia et al. (2022a)
		• Melt blending and solvent casting preparation				
		• Deposition velocity: 300 mm/min				
		• Screw rotation velocity 10–20 rpm				
		• Melting temperature 70–80°C				
	PCL/HA	• SLS technique	Rat, Calvarial Defect (5mm diameter)	VEGF	• Enhanced blood vessel formation	Liu et al. (2020b)
					• Enhanced *in vivo* bone regeneration	
	PCL/HA	• FFF techniquepore size 400 μm	-	-	• Young’s modulus has been increased by 50%	Rezania et al. (2022a)
		• nozzle size 0.4 mm			• Biocompatibility, Alkaline phosphatase (ALP) activity, and calcium deposition all improved after HA was added	
		• nozzle and ambient temperatures: 180 °C and 25°C				
	PCL/nHa	• Extrusion-based technique	-	-	• The best printability, lowest swelling/degradation, and consistent mechanical qualities come via melt blending ink preparation	Zimmerling et al. (2021)
		• Melt-blending, Powder blending, Liquid solvent technique, and Solid solvent technique			• Powder blending offers the best mechanical qualities; however, they are variable	
		• Strand diameter: 0.510 mm and a strand spacing: 1.0 mm				
		• Layer height: 80% of strand diameter				
	PCL-HA	• FDM techniqueDiameter 300 μm	Rabbit, femoral condyle defect (6 mm diameter* depth of 6.5 mm)	Heparan sulfate	Optimal concentration of heparan sulfate increased Biocompatibility, promoted osteoblast maturation and new bone formation, and high compression resistance	Liu et al. (2020a)
		• Pore size400 μm, 20 layers			high concentration of heparan sulfate inhibited (500 μg/mL) osteoblast maturation	
	PCL/HA/CNT	• Extrusion-based technique	-	-	• Addition of 0.75% CNT increased the compressive yield stress (6.5 MPa)	Goncalves et al. (2016)
		• Liquid solvent technique				
		• Pore size: 450–700 µm			• 2 wt% CNT scaffold has the best mechanical and electrical properties	
		• Needle diameter: 0.45 mm			• HA/CNT improve protein adsorption, cell adhesion, and bioactivity	
	PCL/β-TCP	• Extrusion-based technique	-	-	• Improved osteogenic differentiation and expression of related gens and proteins	Park et al. (2018a)
		• Melt-blending				
		• Nozzle diameter:300 μm				
	PCL/β-TCP	• Extrusion-based techniqueNozzle diameter: 400 μm	Dogs, mandibular defect (10.0 × 5.0 × 5.0 mm)	rhBMP-2	• Increased ALP activity, mineralization, new bone formation, and biodegradability	Park et al. (2021a)
		• Scaffolds dimensions: 10 × 4 × 4 mm^3^				
	PCL/βTCP	• Extrusion-based technique	Pigs, Mandibular defects (2 cm/2 cm)	Porcine bone marrow progenitor cells (pBMPC)	• Increased new bone formation	Konopnicki et al. (2015)
		• Micropore (5–40 μm) and macropore (70–300 μm)				
	PCL/β-TCP	• FDM technique	-	-	• Increasing the β-TCP can increase the surface roughness, osteogenic differentiation, and degradation rate	Bruyas et al. (2018a)
		• Solvent Casting preparation			• Increasing the β-TCP can decrease the contact angle	
		• Printing speed:5 mm/s			• Increasing the β-TCP and decreasing the scaffold porosity can increase the Young’s modulus	
		• Printing temperature: 160°C				
		• Struts width: 350μm–400 μm				
	PCL/β-TCP	• FDM and Melt-electrowriting	-	-	activity, and calcium deposition are all improved.A fine fiber grid inserted in the pores of thick fibers can guide cells across bridges and cover the pores.	Wang et al. (2021)
		• Solvent Casting preparation				
		• Cross-scale scaffold (coarse fiber mesh (500 μm), and fine fiber meshes (10 μm))				
	PCL/pristine graphene	• Extrusion-based technique	-	-	• Improved cell viability, proliferation, and hydrophilicity	Wang et al. (2016a)
		• Melting temperature 90°C, slice thickness 220 µm, deposition velocity 20 mm/s				
	PCL/graphene oxide	• Extrusion-based	-	-		Unagolla and Jayasuriya (2019)
		• Solvent Casting preparation			• Cell attachment, proliferation, ALP activity, and mineralization were increased by adding a small amount of GO	
		• 5 × 5 mm mesh, pore sizes (400 μm, and 800 μm), 22 layer, nozzle size: 0.159 mm, temperature 100 °C			• Smaller pore size showed a higher compressive modulus	
		• Speed 1 mm/s, printing pressure 80–100 PSI				
	PCL/Ca-polyphosphate-microparticles	• Eextrusion-based technique	-	-	• Improve cell attachment, bone remodeling, and hydroxyapatite deposition	Neufurth et al. (2017)
		• Powder blending				
		• Temperature 100 C, scaffolds 10 mm diameter * 1.5 mm height, layer thickness 320 mm				
		• Pressure 7.8 bar, speed 3 mm/s				
PCL/polymer/ceramic composites	PCL/β-TCP/Porcine-bdECM	• Extrusion-based technique	Rats, calvaria defect (8 mm diameter)	rhBMP-2	PCL/β-TCP/bdECM/BMP scaffold improved bioactivity, new bone formation, and cell adhesion	Bae et al. (2018b)
		• Melt-blending and Liquid solvent technique				
		• Line width: 300 µm, pore size: 400 µm, and line height: 100 µm				
		Scaffold dimensions: 7 × 7 × 1 mm^3^				
	PCL/bdECM/β-TCP	• Extrusion-based techniqueNozzle size: 500 µm	Rat, calvarial defects (8-mm diameter)	-	• Combination of PCL/bdECM/β-TCP improve osteogenic potential and reduced inflammatory responses	Yun et al. (2021b)
		• Temperature 110°C, and a pneumatic Pressure 550 kPa				
		• Line width 300 µm				
		• Pore size 300µm, layer height 100 µm (4 layers)				
	PCL/β- TCP/Porcine-Derived-bdECM	• Extrusion-based technique	Rabbits, skull defect, (8 mm diameter)	-	• Improved osteogenic potential, cell proliferation, new bone formation	Kim et al. (2018)
		• Melt blending and coating				
		• Temperature 120 °C, pneumatic pressure 500 kPa, scaffold dimension 8 mm diameter * 2 mm height, line width 300 μm				
		Pore size 300 μm, and line height 100 μm				
	Magnesium calcium silicate/gliadin/PCL	• Extrusion-based technique	Rabbits, femoral defects (5 mm)	-	• Addition of magnesium calcium silicate and gliadin improved the compressive strength, cell attachment, cell proliferation, new bone volume, and degradability	Zhang et al. (2018b)
		• Liquid solvent technique				
		• Temperature 130°C, speed 100 mm/min, nozzle size 0.33 mm, line width 500 μm				
		• Pore size 500 μm, line height 500 μm				
	PCL/PLGA/-TCP	• Extrusion-based technique	Rabbits, calvarial defects (8 mm)	rhBMP-2		Shim et al. (2014)
		• Melt-blending and Liquid solvent technique			• Improve new bone formation	
		• Circular scaffold (10 mm diameter)			• Sustained drug release	
		• Head1:temperature 135C and pressure 650 kPa, head 2: temperature 20C and pressure 30 kPa				
	PCL/PLGA/-TCP	• Extrusion-based techniqueTemperature 135C and pressure 650 kPa	Beagle, edentulous mandibular alveolar ridge		• Conserved mechanical properties in wet and dry conditions	Won et al. (2016)
		• Strut width 300 μm, pore size 200 μm, porosity 40%			• Appropriate biocompatibility and bone regeneration (similar to collagen membrane)	
	PCL/hydrogel (alginate/gelatin/nano-hydroxyapatite)	• FDM technique	-	hMSC	• Improved bioactivity, cytocompatibility, mineralization, and osteoconductivity	Hernandez et al. (2017a)
		• Melt-blending			• In comparison to 3D printed mesh and honeycomb scaffolds, the printed gyroid scaffold of PCL enabled for a greater amount of hydrogel to be loaded within the scaffolds	
		• Nozzle size: 0.4 mm, temperature 110C, speed 90 mm/s				
	PCL/Gelatin/Bacterial Cellulose/Hydroxyapatite	• FDM technique	-	-	• Improve cell attachment and proliferation	Cakmak et al. (2020)
		• Liquid solvent technique			• Both the optimal pore size for bone tissue engineering and a uniformity ratio of more than 90% are found in the 80 percent infill rate	
		• Pore size 300 μm, nozzle size 0.5mm, flow rate 0.2 mL/h, 10 Layers, Platform Temperature 38C				
	PCL/TCP/methacrylated hyaluronic acid/methacrylated gelatin	• Extrusion-based technique	Rat, mandibular defect (4 mm)	Resveratrol and Strontium ranelate	Resveratrol had a more sustained release profile, while Strontium ranelate had an initial burst release then a sustained release	Zhang et al. (2020)
		• Liquid solvent technique			• SrRn increase cell proliferation and osteogenic potential	
		• Scaffold 20 mm × 20 mm × 1 mm			• Scaffolds decrease osteoclast activity	
PCL/polymer composites	PCL/polyaniline	• Extrusion-based technique	-	-	• Polyaniline increase the scaffold conductivity	Wibowo et al. (2020)
		• Melt compounding			• Scaffold with 0.1% wt. polyaniline has the suitable conductivity and mechanical properties for bone healing	
		• Nozzle size 330μm, speed 20 mm s^−1^, temperature 90°C, pressure 6 bar			• 1% and 2% wt. polyaniline has cytotoxic effect	
	Pcl/GelMA	• Extrusion-based technique	-	Dental pulp stem cells	• Improve compressive modules to human trabecular bone	Buyuksungur et al. (2021a)
		• Melt-blending and Liquid solvent technique			• 90% cell viability	
		• The printing platform temperature 10C			• Improved osteogenic differentiation and mineralization	
		• Head 1: speed 10 mm/s and pressure 1.8 bar				
		Head 2: speed 2 mm/s and pressure: 7.3 barnozzles size: 400 μm				
	levan/polycaprolactone/gelatin	• FDM technique	-	-	• Levan increase biocompatibility	Duymaz et al. (2019)
		• Liquid solvent technique			• Levan decrease surface tension and compressive strength of the scaffolds	
		Nozzle size 0.4 mm, the build plate temperature 45°C–55 °C, travel speed 100–150 mm.s^−1^				
	PCL/gelatin	• Extrusion-based technique	-	-	• Gelatin improve hydrophilicity, pore size distribution, interconnectivity, and osteogenic differentiation	Azarudeen et al. (2020)
		• Liquid solvent technique				
		• Strut size 0.4 mm, temperature 24 °C, pressure 2.5 bar, speed 30 mm/s				
	PCL/fish bone extract	• Extrusion-based technique	-	-	• Improved cell proliferation, osteogenic differentiation, and calcium deposition	Heo et al. (2019a)
		• Melt blending and coating				
		• Temperature 110C, nozzle size 400 μm				
		• Pressure 500 kPa, velocity 0.2 mm/s				
PCL/metal composites	PCL/Mg	• FDM technique	Rabbits, medial tibial tubercle defect (6 mm diameter *4.5 mm depth)	-	• Biomineralization, biocompatibility, new bone formation, and biodegradability were all increased by magnesium	Dong et al. (2021)
		• Temperature 160°C, speed 1.5 mm⋅s^-1^			• PCL-based scaffolds had the best bone healing ability, mechanical features, biocompatibility, osteogenic potential, and angiogenic capabilities when combined with 3 wt% Mg	
	PCL/Mg	• FDM technique	Rat, skull defect (8 mm)	-	• PCL/10% Mg improve hydrophilicity, cell proliferation, osteogenic activity, new bone formation	Zhao et al. (2020a)
		• Scaffold dimension 8 mm diameter* 1.5 mm height, wire diameter 200 μm				
		• Temperature 110 °C, pressure 0.6 MPa, speed 6–8 mm/s				
	PCL/Zn	• FDM technique	Rats, calvaria defect (6 mm)	-	• Zn improved mechanical properties and cytocompatibility	Wang et al. (2022d)
		• Melt- compounding (filaments fabrication)				
		• Nozzle size 300 μm , speed 500 mm/min, layer thickness 300 μm, pore size 300 μm, porosity 50%			• 2 wt% Zn improved osteogenic potential	
					• increase of Zn can increase osteoclastogenesis	

### 3.1 PCL/ceramic composites

Ceramics are generally hard, wear-resistant, oxidation-resistant, thermal-resistant, inert, and brittle materials with low tensile strength and high compression strength (Jonathan Black, 1998; Farid, 2019). PCL/ceramic composite materials possess a higher level of mechanical properties, bioactivity, hydrophilicity, and biodegradability compared to pure PCL (Fathi-Achachelouei et al., 2019; Biscaia et al., 2022b). Hydroxyapatite (Biscaia et al., 2022b), carbon nanotubes (CNTs) (Gonçalves et al., 2016), β-tricalcium phosphate (β-TCP) (Park H. et al., 2018), graphene (Wang et al., 2016b; Zhang et al., 2018a) and mesoporous bioactive glasses (Zhang et al., 2018a) are the most commonly used ceramic materials in combination with PCL.

**FIGURE 4:**
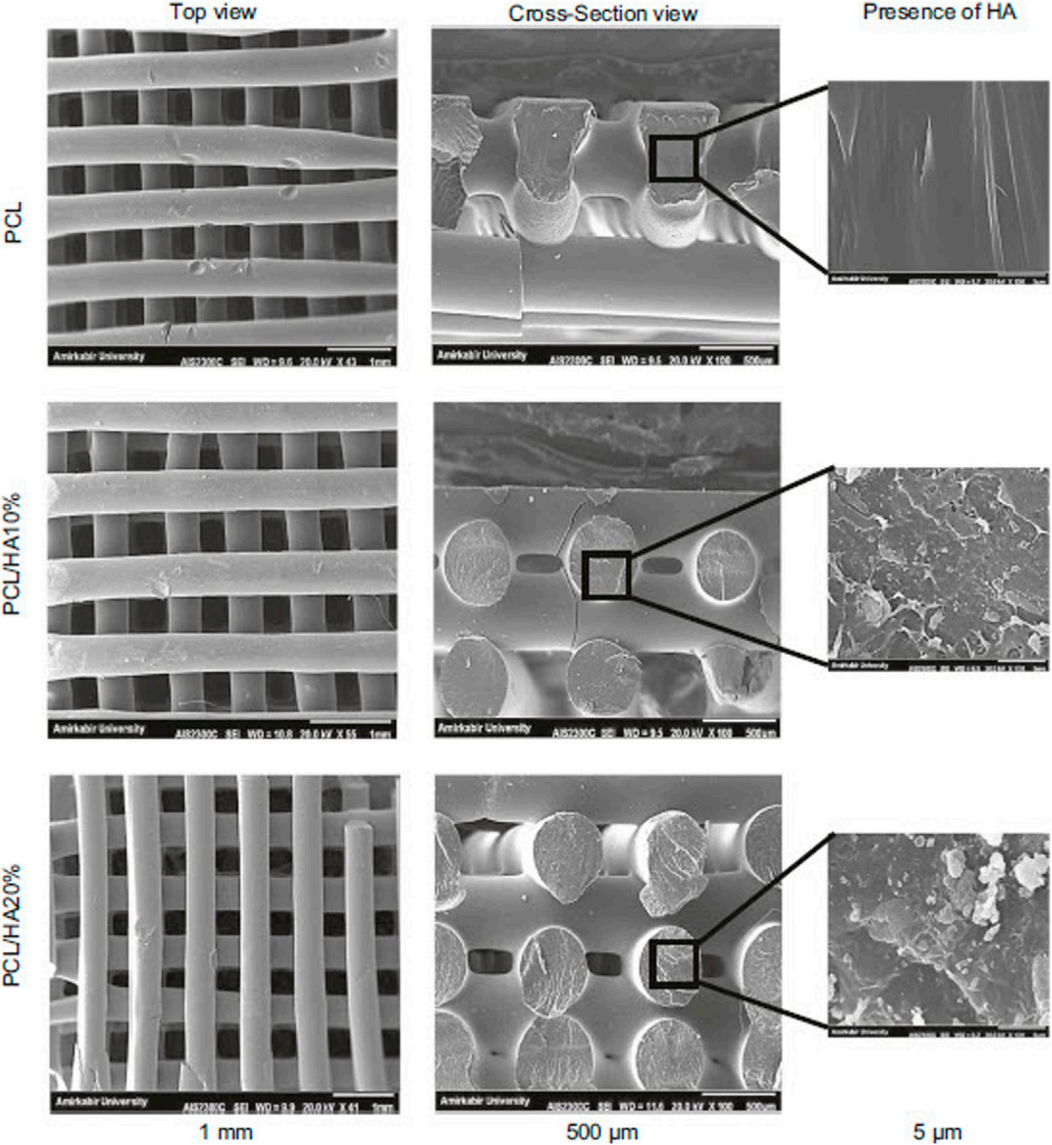
SEM images of PCL composite containing various percentages of HAp, reprinted with permission from (Rezania et al., 2022b).

Hydroxyapatite (Ca_10_(PO_4_)_6_(OH)_2_, HAp) is a ceramic material whose composition and structure are similar to the mineral part of natural bone. This substance plays a pivotal role in strengthening the proliferation of bone cells, increasing protein absorption, improving cell adhesion, and preventing the growth of cancer cells (Hassanajili et al., 2019b; Biscaia et al., 2022b; Rezania et al., 2022b). The addition of HAp to PCL improves biocompatibility, osteoconductivity, bone integration, and the mechanical properties of the composite. HAp has a dense crystalline structure and induces bone matrix mineralization *in vivo* while preventing the formation of fibrotic tissue (Huang et al., 2018; Hassanajili et al., 2019b; El-Habashy et al., 2021). The HAp nanoparticles increase differentiation, bone cell proliferation, mineral deposition, and ultimately accelerate the formation of bone tissues (Figure 4) (Gatto et al., 2021; Zimmerling et al., 2021).

Studies showed that the addition of β-TCP (as an osteoconductive material) to PCL improved its biodegradability (more effective than HAp), mechanical strength, and binding ability of the resulting composite to proteins, growth factors, and cells (Kim Y. et al., 2016; Park H. et al., 2018). The double holes on the TCP surfaces allow the penetration of vascular and bone tissue inside the resulting composite (Tarafder and Bose, 2014; Park H. et al., 2018). Broyas et al. (Bruyas et al., 2018b) investigated the effect of the ceramic material on the properties of the resulting composite by adding 0–60 wt% of β-TCP to PCL. They found that the addition of β-TCP could improve the mechanical properties, biodegradability, and surface properties of the bone scaffold. Huang et al. (Huang et al., 2018) investigated the effect of adding both HAp and β -TCP ceramic materials to PCL. According to their results, the addition of HAp improved the biological and mechanical properties of the resulting composite more than the addition of TCP.

**FIGURE 5:**
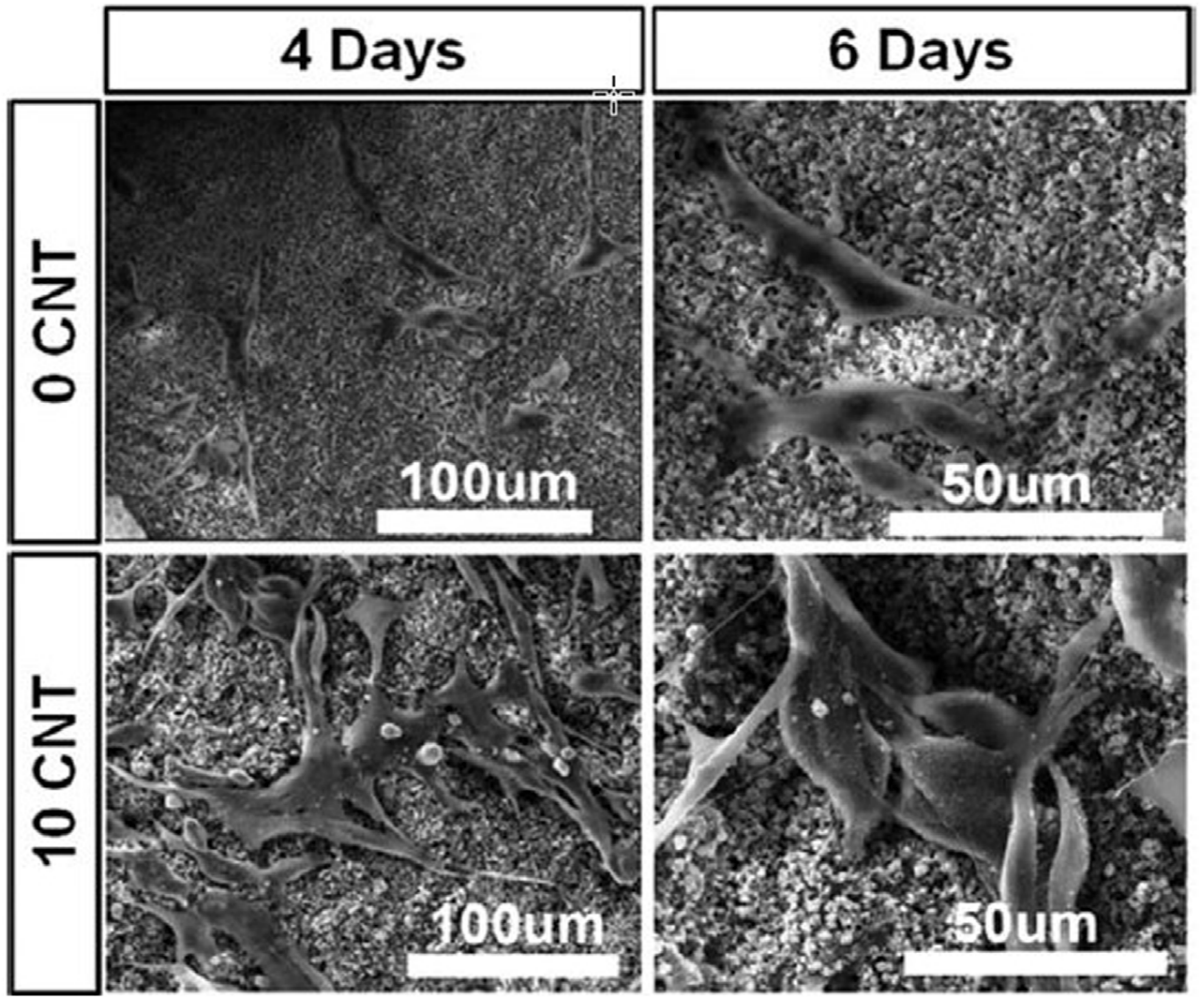
The effect of CNT amount on the attachment of MG63 cells in PCL-CNT composite scaffold, reprinted with permission from (Gonçalves et al., 2016).

CNTs are materials with known chemical, electrical, mechanical, and structural properties, while their combination with PCL improves the electrical conductivity of the resulting composite and induces bone healing (Peidavosi et al., 2022). Carbon nanotubes are often used as imaging agents and carriers of bioactive molecules (Saito et al., 2008; Gonçalves et al., 2016; Wesełucha-Birczyńska et al., 2021). Also, graphene is another ceramic material whose combination with PCL improves its processability, cell attachment, mechanical properties, biological function, and conductivity. However, in terms of cytotoxicity, the use of graphene is challenging, and some studies have shown toxic effects at high doses (Figure 5) (Niyogi et al., 2006; Wang et al., 2016b).

### 3.2 PCL/polymer composites

Polymers are divided into two categories, including synthetic and natural. Semi-crystalline or amorphous synthetic polymers are biodegradable, biocompatible, non-immunogenic, and non-toxic. Synthetic polymers are produced under controlled conditions and thus have tunable mechanical properties, crosslinkability, Young’s modulus, and degradability (Middleton and Tipton, 2000; Hassanajili et al., 2019b). In contrast, natural polymers have higher degradation rates, lower mechanical properties, and better cell binding; however, they have batch-to-batch variations (Bharadwaz and Jayasuriya, 2020). The combination of various natural and synthetic polymers, along with decellularized tissues, with PCL could improve its features.

Polylactic acid (PLA) is one of the most widely used synthetic polymers, which is converted into non-toxic components utilizing a controlled degradation rate. PLA has a more brittle structure, a faster degradation rate, and less flexibility compared to PCL (Hassanajili et al., 2019b). Combining PLA with PCL could overcome the limitations of both polymers, such as britlleness, degradation rate, and cellular attachment efficiency. Xu et al. (Xu et al., 2018) used various composition ratios of PLA/PCL to improve the osteogenic differentiation of hMSCs. By increasing the weight ratio of PLA (up to 80%), the stiffness, bioactivity, and osteogenic potential of the 3D PCL/PLA scaffold were improved. Increasing the weight ratio of PLA led to an improvement in alkaline phosphatase activity and calcium content.

(Poly (lactic-co-glycolic) acid) (PLGA) is a biocompatible and biodegradable synthetic polymer, suffering from poor mechanical properties. The notable fact is that its by-products create an acidic environment (Shim et al., 2015). A study showed that the PCL/PLGA/β-TCP printed scaffold improved bone healing (Hwang K. S. et al., 2017). This composite possessed favorable biological and mechanical properties of PCL and PLGA, along with the osteoconduction feature of β-TCP. The authors emphasized that the 3D printing technique could produce this composite with various thicknesses and pore sizes (Hwang K. S. et al., 2017).

Polyphosphate is an inorganic and physiological polymer found in platelets, serum, and metazoans. This polymer has favorable morphogenetic and biocompatible activity and stimulates anabolic signals and metabolic processes, increasing adenosine triphosphate (ATP) synthesis and hydroxyapatite formation. However, it has poor mechanical properties, and its combination with PCL leads to the creation of a composite with favorable mechanical properties, biocompatibility, and morphogenetic activity for bone tissue repair (Müller et al., 2015; Neufurth et al., 2017).

Gelatin is a natural polymer derived from collagen, which has been used in combination with multiple synthetic polymers, including PCL, due to its low cost, availability, biocompatibility, suitable functional groups, enzymatic biodegradability, and low immunogenicity. Studies have approved that combining gelatin with PCL stimulates osteogenic differentiation and improves the degradation rate of PCL (Buyuksungur et al., 2021b; El-Habashy et al., 2021).

Bone ECM contains a variety of collagens, proteoglycans, growth factors, and non-collagenous proteins; hence, acellular tissues can help in improving intercellular connections, proliferation, and cell differentiation (Bolander and Balian, 1986; Pati et al., 2014). Combining the decellularized bone matrix (bovine or cadaver) with PCL creates a ink that can help promote bone regeneration, cell adhesion, and osteoblast proliferation via accelerating the osteoconductive and osteoinductive signaling procedures (Bae E. B. et al., 2018; Yun et al., 2021a).

**FIGURE 6:**
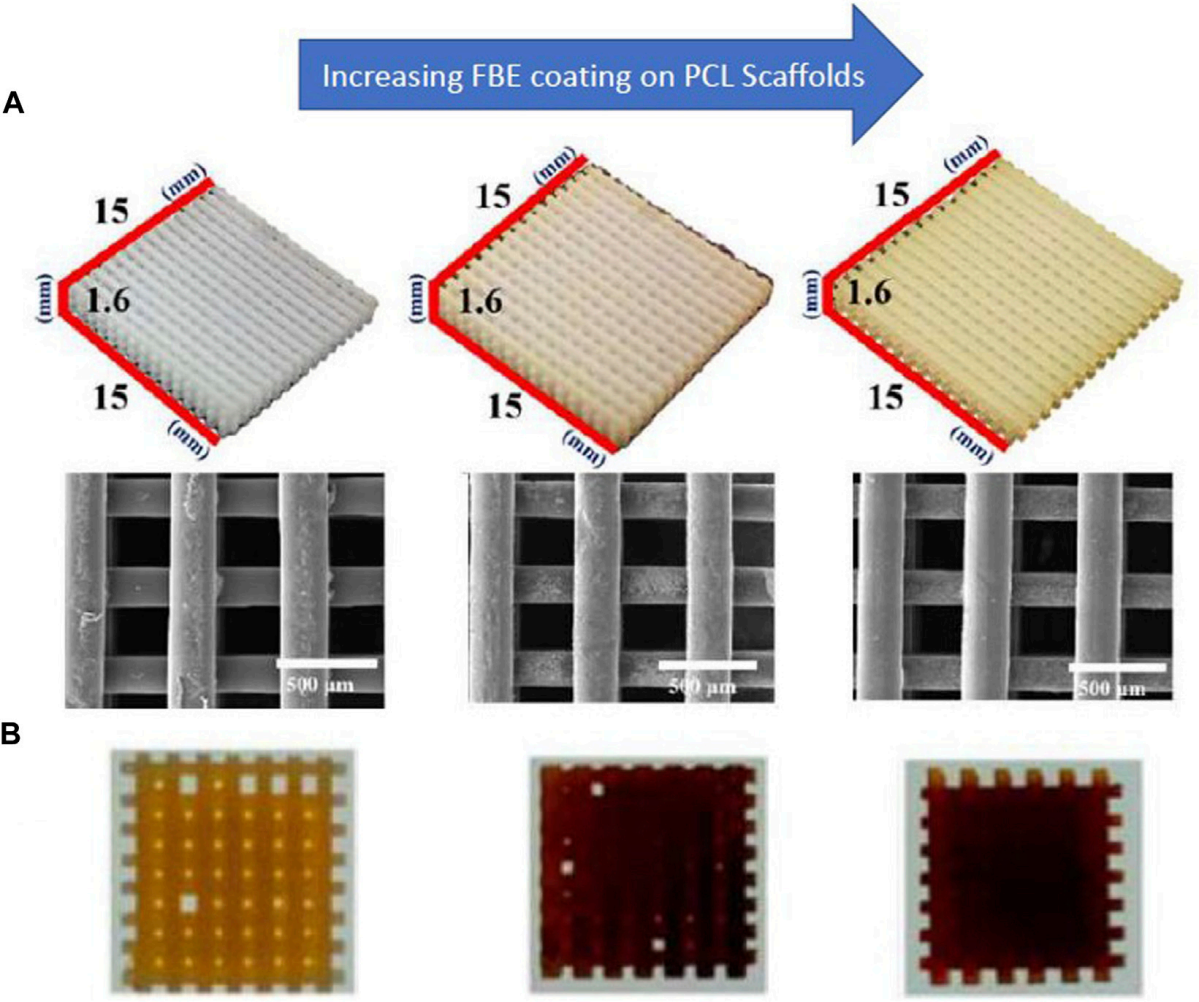
**(A)** SEM images of PCL scaffolds coated with fish bone extract (FBE) **(B)** Alizarin Red staining indicates calcium deposition on the scaffolds, reprinted with permission from (Heo et al., 2019b). The concentration of the FBE coating solutions is 0%, 1%, and 3%, respectively, from left to right.

Not only, the research investigated that fish bone extract has anticoagulant, antibacterial, and antioxidant properties, but also possesses the ability to increase alkaline phosphatase activity (Heo et al., 2019a). The combination of the fish extract with PCL improves osteogenic effects and improves the mineralization of bone tissue (Heo et al., 2018; Heo et al., 2019b). Heo et al. (Heo et al., 2019a) used a coating of fish bone extract on a 3D-printed PCL scaffold to improve its osteogenic properties. Their results showed that the resulting scaffold improved the adhesion and proliferation of MC3T3-E1 cells and promoted the expression of osteogenic genes and calcium deposition (Figure 6). Also, gliadin is a natural polymer of wheat protein with favorable mechanical properties and degradability, and its combination with PCL and bioglass can stimulate the proliferation and differentiation of bovine turbinate fibroblasts and the growth of bone tissue (Reddy and Yang, 2008; Zhang et al., 2018a).

### 3.3 PCL/metal composites

Metals have appropriate strength and toughness, while some challenges, such as stress shielding (mismatching the elastic modulus of metals and natural bone), higher stiffness (decreasing bone density), a slow degradation rate, and the need for a second surgery (to remove excess metal material) have limited their usage. Therefore, the combination of metals with biodegradable polymers such as PCL increases the potential of their application in bone repair (Ciosek et al., 2021; Raj Preeth et al., 2021; Huber et al., 2022). Magnesium (Zhao et al., 2020c), Zinc (Wang S. et al., 2022), and Silver (Shao et al., 2019a) are the most commonly used metals in combination with PCL for bone repair.

Magnesium (Mg) is a biodegradable metal with high mechanical properties and FDA approval (Han et al., 2019). Magnesium is involved in angiogenesis, antibacterial effects, proosteogenic effects, protein synthesis, oxidative phosphorylation, and mineralization of bone tissue (Roh H. S. et al., 2017; Peng et al., 2021). Studies showed that magnesium stimulated the differentiation of osteoblasts (Wang J. et al., 2017). The composition of magnesium in PCL can increase the bioactivity, degradation rate, and mechanical properties of the resulting composite according to the needs of bone tissue (Zhao et al., 2020c).

Zinc (Zn) is a metal with favorable mechanical strength, biocompatibility, and degradation rate, and its decomposition products do not contain hydrogen gas (Maleki-Ghaleh et al., 2021; Wang S. et al., 2022). Zn is involved in energy metabolism, angiogenesis, protein synthesis, collagen synthesis, bone formation, and mineralization (Ghorbani et al., 2015; Li et al., 2019). The combination of Zn and PCL can improve mechanical properties and osteogenesis. Studies have shown that 1–50 μM of Zn is favorable for osteogenesis, and doses of more than 50 μM inhibit osteogenesis (Wang S. et al., 2022).

## 4 Incorporation of cells in scaffolds

To integrate living cells into PCL-based scaffolds during printing, bioinks and bioprinter devices described in Section 2.1 are used. Bioink includes a mixture of several biomaterials (generally in the form of hydrogel) and desired cell types, which are used under specific conditions of bioprinters to create tissue constructs (Borkar et al., 2021). Cell-laden bioinks enable the creation of functional tissues from various cells and biomaterials. The possibility of integrating the patient’s cells into the bioink reduces the risk of transplant rejection (Beheshtizadeh et al., 2022). Cell encapsulation provides homogeneous cell seeding and suitable anchorage, which leads to proper cell signaling, cell function, and tissue repair. The combination of PCL with various cells in the bioprinting process increases the clinical application of the resulting scaffolds (Murphy et al., 2017b; Buyuksungur et al., 2021b).

Buyuksungur et al. (Buyuksungur et al., 2021b) used the combination of PCL and GelMA containing dental pulp stem cells (DPSCs) for BTE applications. The cell-containing hydrogel was printed between the PCL struts. The resulting scaffolds had osteoinduction properties (due to cell-loaded GelMA), sufficient mechanical strength (due to PCL), and a compressive modulus similar to trabecular bone’s modulus. The cells in this structure had a high survival rate (over 90%) and were uniformly distributed. The authors claimed that this scaffold supported bone differentiation and mineral deposition.

Murphy et al. (Murphy et al., 2017b) printed the scaffold composed of PCL and borate glass, which contained human adipose stem cells (ASCs). The separated nozzle was used to print ASCs suspended in Matrigel. While the ASCs possess a high viability, the authors pointed that the angiogenesis rate increased with the increasing borate glass. This bioactive scaffold was introduced for BTE applications. Also, in another study, Hernandez et al. (Hernandez et al., 2017b) used a combination of hydrogel (alginate and gelatin) containing human MSCs with PCL for bioprinting. The scaffold had favorable cytocompatibility, cell adhesion and viability, bioactivity, and the growth of apatite crystals.

Pati et al. (Pati et al., 2015b) created a bone microenvironment by printing PCL/PLGA/β-TCP/decellularized ECM containing nasal inferior turbinate tissue-derived MSCs. Based on the researchers’ report, scaffolds supported the osteoblastic differentiation of cells and enjoyed increased calcium deposition. They reported that the scaffolds increased the expression of RUNX2, osteocalcin, alkaline phosphatase, and osteopontin (about four times).

Moreover, Dong et al. (Dong et al., 2017) encapsulated rabbit bone marrow MSCs and BMP-2 in chitosan hydrogel and then printed the combination of this hydrogel and PCL. The cells had an appropriate survival rate, and a uniform distribution of them was reported in the structure. Furthermore, scaffolds had osteogenic, osteoconductive, and bone matrix formation properties, as well as the mechanical strength necessary for BTE applications.

## 5 Incorporation of angiogenic and osteogenic growth factors in scaffolds

Various drugs and growth factors are integrated into the printed scaffold to increase bioactivity, osteogenesis, angiogenesis, osteoclast prevention, and bone regeneration. In addition, it avoids the issues associated with the local administration of growth factors, such as systemic toxicity, a short half-life at the injection site, and action at aberrant sites (Li et al., 2020b; Liu et al., 2020c). PCL scaffolds allow the incorporation of multiple drugs and growth factors simultaneously. These factors can be coated by physical adsorption (Park et al., 2021b) and diverse chemical agents, or incorporated (Kim S. E. et al., 2016; Bae E. B. et al., 2018) in printed PCL composite scaffolds. Multiple drugs, including aspirin (Li et al., 2020a), alendronate (Kim S. E. et al., 2016), lidocaine (Shao et al., 2019b), resveratrol (Zhang et al., 2020), strontium ranelate (Zhang et al., 2020), levofloxacin (Puppi et al., 2016a), doxorubicin (Zhang et al., 2014b), heparin sulfate (Liu Y. et al., 2020), cefazolin (Lee et al., 2022), rifampicin (Lee et al., 2022), and roxithromycin (Bai et al., 2020), along with some growth factors, such as vascular endothelial growth factor (VEGF) (Liu et al., 2020b), and bone morphogenetic proteins (BMPs) (Bae E.-B. et al., 2018), are effective in 3D-printed PCL-based composites.

VEGF is involved in angiogenesis, endothelial cell stimulation, and osteogenesis. Liu et al. (Liu et al., 2020c) modified the surface of PCL/HAp-printed scaffolds by VEGF and apatite coprecipitation. Their results showed that the VEGF improved the formation of blood vessels, osteogenic differentiation, and bone regeneration.

BMPs are transforming growth factor β (TGF-β) family members that play an important role in bone regeneration (via the Smad/MAPK pathway) and osteoblastic differentiation (Beederman et al., 2013). In addition, in the immature region of the BMP-7 protein, there is a peptide called bone-forming peptide 1 (BFP-1), which has higher osteogenesis and bone differentiation activity compared to BMP-7 (Li et al., 2020b; Park et al., 2021b). Bae et al. (Bae E. B. et al., 2018) printed PCL/β-TCP composite containing decellularized bone and BMP-2. They reported that the presence of BMP-2 increased bioactivity and bone mass volume. Other studies showed that PCL/β-TCP composite is a better carrier for BMPs than PCL and results in significant bone volume (Park et al., 2021b).

Aspirin is an anti-inflammatory and non-steroidal drug that inhibits osteoclast formation (by inhibiting the nuclear factor kappa-B pathway) and improves bone formation (by increasing the survival of bone marrow MSCs and stimulating the differentiation of preosteoblasts). Aspirin is effective on bone metabolism, and in low doses, it improves mineral density (Chin, 2017; Liu et al., 2022). The combination of aspirin and basic fetoprotein (BFP) in the PCL scaffold printed by Li et al. (Li et al., 2020b) improved osteogenic differentiation, bone remodeling, and the amount of new bone formation.

Platelet-rich plasma with various growth factors such as VEGF, platelet-derived growth factor (PDGF), and TGF-β improves the proliferation and differentiation of stem cells and bone repair. Li et al. (Li et al., 2017) coated the printed PCL scaffolds with platelet-rich plasma and enhanced the expression of RUNX2, osteocalcin, alkaline phosphatase, and osteopontin genes. The authors reported that these scaffolds improved bone formation and osteogenic differentiation.

The family of bisphosphonates, such as alendronate, are mainly nitrogen-containing drugs and play an essential role in osteoporosis treatment (Liberman et al., 1995). Alendronate inhibits the synthesis of the substance needed to stimulate osteoclasts (geranylgeranyl pyrophosphate) and prevents bone resorption. In addition, improving the mechanical connection of the scaffold with the surrounding tissue accelerates bone healing (Tarafder and Bose, 2014; Kim S. E. et al., 2016). Alendronate increases the expression of BMP-2, osteocalcin, collagen, osteopontin, and alkaline phosphatase (Kim S. E. et al., 2016). Kim et al. (Kim S. E. et al., 2016) printed PCL/alendronate scaffolds, and reported that the sustained-alendronate release increased alkaline phosphatase activity, mineralization, and bone formation.

Shao et al. (Shao et al., 2019a) printed the PCL/Ag_3_PO_4_/lidocaine composite to benefit from the antibacterial effects of Ag_3_PO_4_ and lidocaine as an analgesic. Their results showed that it is possible to control the release of lidocaine by changing the diameter of the printed PCL filaments, so that it reaches the therapeutic effect within 4–7 days. In addition, by changing the amount of Ag_3_PO_4_-loading, their release can be controlled, and a sufficient antibacterial level can be achieved in at least 6 days. Levofloxacin (an antibiotic)/PCL was printed by Pupp et al. (Puppi et al., 2016b) to prevent the infectious complications associated with scaffold implantation with its controlled release. Their results showed a stable and uniform drug release profile during the 5 weeks.

## 6 Clinical applications

The controllability and unique properties of PCL, 3D printing technology, and the results of various animal models indicate the potential of this complex for clinical evaluations. This bioabsorbable polymer has been registered since 1980 and has resulted in potentially non-toxic products in diverse tissues (Moers‐Carpi and Sherwood, 2013). Furthermore, PCL has been used in various clinical evaluations for bone tissue repair (available: https://beta.clinicaltrials.gov/). Clinical trials are conducted according to “good clinical practice” (GCP) standards in 3 phases. The product’s safety and effectiveness are evaluated on a small number of patients in the phases I and II, respectively. And the phase III, with the increase in the number of patients and the confirmation of the product’s effectiveness, began its commercialization process (de Vries et al., 2008; Hollister and Murphy, 2011). The selection of patients for clinical trials should be done according to a specific protocol to be approved by regulatory centers and to reduce the influence of various factors on the results. Patient safety must be prioritized at all stages and must not pose a severe risk to the patient (Kleiderman et al., 2018; Kumar Gupta et al., 2022).

In a clinical trial, PCL was used to help improve and prevent the reduction of alveolar ridge height after tooth extraction, because the reduction of alveolar ridge height and volume prevents implant placement. This clinical evaluation was performed on 13 patients in two control groups (without scaffold) and the group with PCL scaffold immediately after tooth extraction. The width and height of the alveolar ridge were investigated 6 months after tooth extraction. Their results showed that the PCL scaffold improved bone healing and the maintenance of ridge height after 6 months (Tin Goh et al., 2014).

In another clinical trial, printed PCL scaffolds were used to repair caudal septal deviations. These printed scaffolds were used in 20 patients undergoing septoplasty, and the patients were followed up for 12 weeks. Their results showed that PCL scaffolds had favorable mechanical properties, biocompatibility, and ease of surgical manipulation. In addition, the presence of scaffold improves the Nasal Obstruction Symptom Evaluation score, the minimum cross-sectional area and the volume of the nasal cavity changes, and the nasal septum angle changes (Yun et al., 2018). In addition, PCL impregnated with Platelet-rich fibrin (PRF) has been used for ten outpatients with insufficient alveolar bone height around dental implants. Patients were follow-up for 3 months. Their results showed that this combination helped repair bone defects around dental implants and increase bone volume. In addition, no side effects (implant movement, pain, or infection) were observed after 3 months (Verma et al., 2014). Interbody fusion cage for the lumbar spinal stenosis treatment is another case that has used 3D printed PCL/TCP for clinical evaluations. In Xijing Hospital, 22 volunteer patients aged between 30 and 85 years underwent posterior lumbar interbody fusion surgery with PCL/TCP cages and were follow-up for 12 months. Their results showed that this cage increased bone fusion by 95.2%. And a significant improvement in clinical results was observed. Of course, entering the clinic requires longer evaluations and more patients (Liu et al., 2023). Other clinical trials have also used PCL for bone repair; some of these are mentioned in Table 3. However, it is necessary to mention that bed-to-bedside translation of these products requires overcoming problems related to ethical issues, cost, regulatory rules, and ease of use by physicians (Hollister and Murphy, 2011).

**TABLE 3:** Clinical trials performed with PCL scaffolds for bone repair (database, 2018).

Composition	Indication	Clinical phase	Location
PCL	Orbital Fractures	Phase 2	Singapore/2004
3D-Printed PCL	Cranioplasty	Not Applicable	Singapore/2006
PCL/PRP/rhBMP-2	anterior mandible defect	Not Applicable	Germany/2009
PCL/TCP	Orbital walls	Phase 2	Singapore/2010
PCL	Nasal Septal Deformities	Not Applicable	Korea/2016
PCL mesh	orbital floor fracture	Not Applicable	Singapore/2017
3D Printed PCL-TCP	Ridge Preservation AfterTooth Extraction	Not Applicable	Singapore/2019
Bone Marrow Aspirate Concentrate+3D-Printed PCL	Alveolar Defects	Not Applicable	Egypt/2022
3D-Printed PCL	pectus excavatum defects	Not Applicable	Australia/2022
PCL/PRF	Increasing bone volume and quality for dental implant implantation	Phase 1	India/2014
3D Printed PCL-TCP	lumbar interbody cage	Not Applicable	China/2023
PCL membrane	guided bone regeneration	Not Applicable	Thai/2020

The biggest challenges of the 3D printed PCL-based scaffolds to transfer to the bedside included the cost of printers, materials, and pre-and post-processing operations. These costs must be at least equivalent to current expensive treatments for scaffolds to be commercially viable. Although the cost of 3D printers has decreased significantly in recent years, it is still expensive for many medical centers. In addition, the cost of 3D printing materials is often higher than that of traditional methods (Li et al., 2015).

The lack of trained personnel is another challenges of transferring 3D printers to the bedside. Although the technology is becoming more user-friendly, it still requires trained professionals who can work with printers and design patient-specific scaffolds. In addition, the lack of accurate knowledge among doctors about this process and the lack of detailed studies on the effectiveness and safety of these products have led to the fact that doctors are more inclined to use traditional methods. Close communication between clinicians and researchers will help accelerate the translation of this approach to the clinic (Hollister and Murphy, 2011). Furthermore, new technology such as, machine learning is a subset of artificial intelligence, which in recent years, with the help of 3D printing, has significantly reduced the time, cost, and effort required to design patient-specific models from imaging data (Huff et al., 2018).

Regulatory issues will also play an important role in the future evolution of 3D-printed PCL-based scaffolds at the bedside. Because 3D printing is a relatively new technology, guidelines for evaluating the safety and efficacy of 3D printed scaffolds are constantly evolving (Li et al., 2015). The FDA actively tracks the clinical 3D printing industry and has published precise guidelines for 3D printer manufacturers. There are several regulatory challenges for 3D-printed PCL-based scaffolds (especially cell-containing scaffolds). Generally, scaffolds without cells are known as class II medical devices, and scaffolds containing cells are known as class III medical devices in the guidelines, each of them requires compliance with certain standards and controls. Therefore, it is necessary to provide a specific regulatory path and standards to ensure the safety and reproducibility of 3D-printed PCL-based scaffolds (Li et al., 2015; Rahman et al., 2018; Bliley et al., 2022).

Although there are standards such as ISO/DIS 17296–1 for 3D printing terminology and 3D printer manufacturers, there is currently no specific standard for 3D bioprinting technology and inks (ISO, 2012). The level of standardization of inks and process is effective on the product development time and accelerates the clinical translation of the product, controls its quality, and reduces costs. In fact, with standardization, the manufacturers can optimize the printing method and ink composition and minimize the resources needed to make the product (Murphy et al., 2020; Jin et al., 2022).

Logistics requirements are another challenge in the clinical transfer of these products. Especially scaffolds containing cells are environmentally and time sensitive and require the design of a central logistics chain to ensure data collection and transplant tissue production. The patient-derived cells and materials must be transported to a center, and the printed scaffolds returned to the patient (Murphy et al., 2020).

## 7 Conclusion and future perspectives

PCL, a thermoplastic and biodegradable polymer with unique mechanical properties and a slow degradation rate, is a potential biomaterial for the fabrication of BTE scaffolds. The compatibility of this polymer with 3D printing technology provides the patient-specific scaffolds with the desired size, shape, porosity, chemical composition, and suitable dimensions for the target tissue. Considering the better performance of PCL composite scaffolds compared to pure PCL, recent studies have used the combination of various ceramic, polymer, and metal materials with PCL to make bone tissue engineering scaffolds. The integration of these materials improves the properties of cell adhesion, degradability, osteoinductivity, and angiogenesis of the composite and helps to accelerate bone healing. In addition, various studies have also used the integration of drugs and growth factors to improve osteogenesis and angiogenesis in these scaffolds. 3D printing technology can integrate cells into PCL composite scaffolds. PCL composite scaffolds have been used in limited clinical trials to repair alveolar, orbital walls, and nasal septal defects, which indicates the potential of these composites for future applications. Of course, most of these trials are in their initial phases. Therefore, more clinical trials should be conducted for the design, implementation, and scalability of these composites. Integrating 3D printing technology with PCL composites soon will make it possible to create customized composite scaffolds containing the patient’s cells by better mimicking bone architecture to aid in faster bone healing.
